# Can Low-dose Tranexamic Acid Decrease Blood Loss and Transfusion Requirements in Total Knee Arthroplasty?

**DOI:** 10.7759/cureus.2640

**Published:** 2018-05-17

**Authors:** Ozgur Senturk

**Affiliations:** 1 Anesthesiology and Reanimation, Maltepe University Faculity of Medicine, istanbul, TUR

**Keywords:** tranexamic acid, total knee arthroplasty, blood transfusion

## Abstract

Introduction

Tranexamic acid (TA) has been used successfully in primary total knee arthroplasty (TKA) to minimize blood loss and transfusions. The aim of this study was to evaluate the effect of perioperative single low-dose TA administration on postoperative blood loss and necessity for blood transfusion in patients undergoing total knee arthroplasty under spinal anesthesia.

Material and Methods

Data of patients undergoing TKA between January 2013 through December 2015 were retrospectively reviewed. Patients that underwent unilateral knee arthroplasty under spinal anesthesia were separated into two groups: those where TA was used (10 mg/kg) and a control group where TA was not used. The following data were collected from medical records and compared between groups according to demographics, hemoglobin levels, and drainage amount.

Results

A total of 48 patients' data were evaluated (TA: 20; control: 28). The use of tranexamic acid in total knee arthroplasties seemed to reduce the postoperative drainage rate and blood transfusion requirement. Average 24-hour drainage levels were significantly lower in the TA group when compared to the control group. Required 24-hour total blood transfusion amounts were significantly higher in the control group. There was no difference in preoperative and postoperative sixth or 24th-hour hemoglobin levels between the groups.

Conclusion

A low or single dose of TA is a safe and effective agent that significantly lowers blood loss and allogeneic blood transfusion requirements.

## Introduction

Orthopedic surgery, especially that involving spinal surgery or arthroplasty, is associated with excessive bleeding and high rates of transfusion [1-2]. The rate of allogeneic red blood cell transfusion is reported to be 21 - 70% in routine hip and knee arthroplasties and postoperative drainage and hidden blood loss are reported to be around 1,500 - 1,900 ml after total knee arthroplasties [3-4]. Transfusions have the potential risks of ABO incompatibility, immunosuppressant leading to infection, increased procedure costs, and long hospital stay [5-6].

Tranexamic acid (TA) is a synthetic analog of the amino acid lysine [7], which inhibits fibrinolysis by blocking the effect of plasminogen [8-10]. The use of TA in total hip and knee arthroplasties is reported to decrease the perioperative loss of blood by 10 - 150 mg/kg [11-12]. Many studies of different application sites, various doses, and application methods (bolus and/or infusion) have demonstrated that used alone or in addition with other drugs, the application of TA decreases the amount of bleeding and transfusion-associated morbidity and mortality rates [3, 13-18].

The aim of this study was to evaluate the effect of a preoperative routine 10 mg/kg single dose TA administration on postoperative blood loss and the necessity for blood transfusion in patients undergoing total knee arthroplasty under spinal anesthesia.

## Materials and methods

After institutional review board approval, the medical records of all patients undergoing total knee arthroplasty between January 2013 and December 2015 were retrospectively reviewed.

Patients that underwent unilateral knee arthroplasty under spinal anesthesia were separated into two groups: those where TA was used and a control group where TA was not used. The following data were collected from the medical records and compared between groups: age, gender, weight, body mass index (BMI), American Society of Anesthesiology (ASA) score, operation time, tourniquet time, hemoglobin (Hb) levels, mean arterial pressure measurements at postoperative sixth and 24th hours, the total 24-hour drainage amount, transfusion requirement within 24 hours after surgery, visual analogue scales (VAS), and pre- or postoperative complications (thromboembolic events, acute renal failure, myocardial ischemia, allergy, transfusion reactions, etc).

All procedures were performed by the same surgeons. Beginning in January 2013, patients undergoing knee arthroplasty were given a single dose of 10 mg/kg TA (Transamine®, Bilim Ilac, Turkiye) intravenously, half an hour before the opening of the tourniquet. Hemoglobin levels of patients were routinely measured preoperatively and at postoperative 6th and 24th hours. A preoperatively placed drain was removed at the postoperative 24th hour, and drainage amount was noted. All patients routinely received intravenous patient-controlled analgesia for pain control and low molecular weight heparin for thromboembolic prophylaxis. Patients were followed up at outpatient settings four to six weeks after surgery, whereas any postoperative complications were noted. The transfusion indications at our institute for patients included in this study were:

1.  Hb < 8 mg/dl in patients without coronary artery disease or Hb < 10 mg/dl in patients with coronary artery disease or systemic diseases;

2.  Physiological findings of inadequate oxygenation (myocardial ischemia, hemodynamic instability, etc.);

3.  Sudden decrease of Hb level postoperatively or the presence of syncope, weakness, or palpitations, plus the sudden increase in the drainage amount.

Patients with the following were excluded from this study: history or presence of coagulopathy or bleeding disorder, renal dysfunction, use of anticoagulants, acute infection, coronary artery disease, history of deep vein thrombosis, pulmonary embolus, cerebrovascular event, TA allergy, preoperative Hb < 8 mg/dl, patients undergoing bilateral arthroplasty or arthroplasty revision, general anesthesia, and combined spinal-epidural or epidural, plus peripheral nerve block. Patients undergoing general anesthesia were excluded as the results of this study may have been affected by comorbidities more frequently observed in patients undergoing general anesthesia.

All of the statistical analysis was performed using the Number Cruncher Statistical System (NCSS) 2007 (NCSS Statistical Software, Kaysville, Utah, USA). In addition to descriptive statistics (mean, standard deviation, median, frequency, rate, minimum, and maximum), variables with normal distribution were compared using Student’s t-test and variables with non-normal distribution were compared using the Mann Whitney U Test. For intergroup comparison of variables with normal distribution, Repeated Measures Test and Variance Analysis was used. Additionally, the comparison of two sub-groups was performed using the Bonferroni Correction Post Hoc Test. Intergroup analysis of variables with non-normal distribution was performed using the Friedman Test and the comparison of two sub-groups was performed using Wilcoxon Signed Ranks Test. Qualitative data were compared using Fisher’s Exact Test and Yates’ Continuity Correction Test. A value of p < 0.05 was considered as statistically significant, while p < 0.01 was considered as extremely significant.

## Results

One hundred and ninety-two patients’ medical records were reviewed. After inclusion and exclusion criteria were considered, 48 patients with an average age of 67.31 ± 7.22 (47 - 80) years (85.4% female and 14.6% male) were included in this study (Table 1).

**Table 1:** Evaluation of the Demographic Characteristics Within Groups ^a^Student's t-test; ^b^Fisher’s exact test; ^c^Yates continuity correction test; *p < 0.05; **p < 0.01 ASA: American Society of Anesthesiology; BMI: body mass index; hb: hemoglobin; n: number; SD: standard deviation

			Groups		P
		All Patients	Tranexamic acid (n = 20)	Control (n = 28)	
		Mean ± SD	Mean ± SD	Mean ± SD	
Age (years)		67.31 ± 7.22	69.35 ± 5.89	65.86 ± 7.82	^a^0.099
Weight (kg)		79.10 ± 14.16	74.05 ± 11.80	82.71 ± 14.79	^a^0.035*
BMI (kg/m^2^)		30.90 ± 5.53	28.93 ± 4.61	32.31 ± 5.78	^a^0.035*
		n (%)	n (%)	n (%)	
Sex	Female	41 (85.4)	16 (80.0)	25 (89.3)	^b^0.429
	Male	7 (14.6)	4 (20.0)	3 (10.7)	
ASA	II	24 (50)	8 (40.0)	16 (57.1)	^c^0.380
	III	24 (50)	12 (60.0)	12 (42.9)	

**Figure 1:**
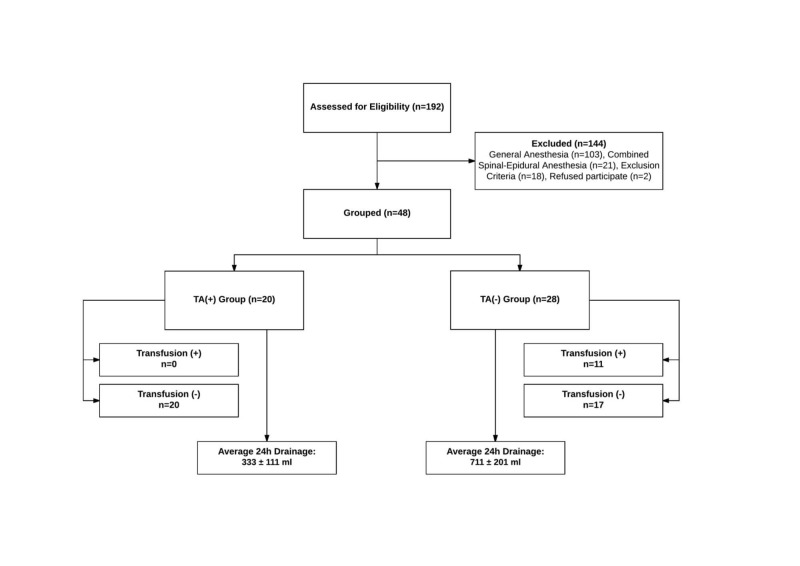
Flow chart of study for tranexamic acid (TA) n: number

The flowchart diagram for study inclusion is given in Figure 1.

Surgical time ranged from 90 - 180 minutes with an average of 119.69 ± 20.46 minutes. Average tourniquet time was 91.46 ± 13.60 minutes and ranged from 65 – 120 min. Surgical or tourniquet times were similar between the two groups (p > 0.05). There was no significant difference in postoperative sixth and 24th hour Hb levels between the groups (p > 0.05).

The patients’ Hb distribution is shown in Table 2.

**Table 2:** Assessment of Groups According to Hemoglobin (Hb) Levels ^a^Student's t-test; ^b^Yates Continuity Correction Test; ^c^Fisher’s Exact Test; hb: hemoglobin; P: p value; SD: standard deviation

Hb (mg/dl)		Group		^a^p
	All Patients	Tranexamic acid	Control	
	Mean ± SD	Mean ± SD	Mean ± SD	
Preoperative	12.48 ± 1.50	12.47 ± 1.47	12.50 ± 1.54	0.944
Postoperative sixth hour	10.63 ± 1.27	10.75 ± 1.20	10.54 ± 1.33	0.570
Postoperative 24th hour	9.97 ± 1.18	10.15 ± 1.12	9.85 ± 1.23	0.385
	^b^p	0.001	0.001	
Preoperative – Postoperative sixth hour		^c^0.001	^c^0.001	
Preoperative – Postoperative 24th hour		^c^0.001	^c^0.001	
Postoperative sixth hour - Postoperative 24th hour		^c^0.001	^c^0.001	

The average 24-hour drainage amount was 553.85 ± 252.64 ml (170 - 1,080 ml), and 22.9% (n = 11) of patients required a blood transfusion within 24 hours postoperatively. When the two groups were compared, there was no difference between mean arterial pressure or VAS scores at the 6th or 24th postoperative hours (p > 0.05). There was no difference in the average age between groups (p = 0.099; p > 0.05). The patients’ weight and BMI were significantly lower in the TA group (p = 0.035; p < 0.05) (Table 1).           

Patients in the TA group had significantly less amount of drainage within 24 hours when compared to the control group (p = 0.001; p < 0.01), and the blood transfusion requirement of the control group within 24 hours was significantly higher (p = 0.001; p < 0.01) (Table 3).

**Table 3:** Drainage and Total Blood Transfusion Assessment by Group ^a^Mann-Whitney U Test; ^b^Fisher’s Exact Test; n: number; P: p value

		Group		P
		Tranexamic Acid	Control	
		Mean ± SD (Median)	Mean ± SD (Median)	
24-Hour Drainage (ml)		333.00 ± 111.08 (315.00)	711.61 ± 201.20 (695.00)	^a^0.001
		(n = 20) (%)	(n = 28) (%)	
24-Hour Total Blood Transfusion	1 Unit	0 (0.0)	11 (39.3)	^b^0.001
	None	20 (100.0)	17 (60.7)	

When evaluating the demographics of the patients within the control group requiring transfusion and those not requiring transfusion, there was no difference (p > 0.05).

## Discussion

Results of this study have demonstrated that the use of a single dose of TA (10 mg/kg) in patients undergoing total knee arthroplasty under spinal anesthesia decreases 24-hour drainage amounts when compared to controls. There was no difference in the preoperative and postoperative sixth or 24th-hour hemoglobin levels between the groups. However, when patients in the control group were separated into those who received transfusion and those who did not, the preoperative and postoperative 6th and 24th-hour hemoglobin levels were significantly lower in patients who received a transfusion. No patient receiving TA required a blood transfusion, and no perioperative or postoperative complication due to TA was observed.

Postoperative blood loss leading to hemodynamic instability, blood transfusions, and slower healing is an important risk in patients undergoing total knee arthroplasty. Tourniquet use, hypotensive anesthesia, hemodilution, autologous blood donation, preoperative erythropoietin application, blood salvage, and more recently, antifibrinolytic drugs have been used to decrease this risk [12-13, 19].

The surgical procedure in total knee arthroplasty generally involves the use of a tourniquet, leading to less intraoperative but more significant postoperative blood loss [5]. The use of a tourniquet exposes the lower extremity to anaerobic conditions leading to a fibrinolytic reaction and increased postoperative blood loss [20]. Data from our study also showed longer tourniquet times required more blood transfusions.

TA inhibits fibrinolysis by blocking the effect of plasminogen [8-10]. This effect of TA on the coagulation cascade leads to an increased coagulability state, which may increase the risk of pulmonary emboli, deep vein thrombosis, myocardial infarction, and cerebrovascular events [21]. Duncan et al. reported no increase in postoperative venous thromboembolism or 30-day mortality when TA was used in primary or revision total hip or total knee arthroplasties [10]. Shen et al. performed deep vein Doppler ultrasonography one week after surgery in patients undergoing total knee arthroplasty with 15 mg/kg TA administration and found no increased risk of deep vein thrombosis [20]. In a meta-analysis by Yang et al., TA used in patients undergoing total knee prosthesis did not increase the prothrombin time, activated partial thromboplastin time, or change the prevalence of deep vein thrombosis or pulmonary emboli [22].

Various studies and meta-analyses have demonstrated that different dosages and applications of TA decrease perioperative and postoperative blood loss and the requirement for red blood cell transfusion, yet do not have a significant effect on mortality or morbidity [4, 8, 10, 22-24]. Samujh et al. reported that a single dose of intravenous TA (10 mg/kg) significantly decreased the transfusion requirement (p = 0.03) in patients undergoing revision total knee arthroplasty [24]. They found no difference between age, preoperative hemoglobin, hospitalization time, or intraoperative blood loss [24]. In our clinic, we use the recommended lowest dose TA (10 mg/kg) in a single application, due to patient comorbidities, potential venous thromboembolism risk, and the difficulty of performing multiple doses postoperatively.

This study also evaluated the 24-hour drainage amount in patients undergoing total knee arthroplasty. We found that patients who received TA had significantly less 24-hour drainage and total blood transfusion requirements (p < 0.01). Our data have demonstrated that even at the lowest recommended dosage of 10 mg/kg, TA is an effective method. No patient required additional doses.

Various studies have analyzed factors affecting postoperative bleeding and transfusion requirements. Carling et al. found low preoperative hemoglobin levels, low BMI, and long operating times to be correlated with allogeneic blood transfusions in patients undergoing total knee arthroplasties [4]. Whiting et al. demonstrated that the use of TA in patients undergoing total knee arthroplasty significantly decreased transfusion need and hospitalization time and recommended its use in all patients independent of preoperative hemoglobin levels [19]. In a retrospective multicentered study, Poeran et al. reported TA use led to a 69% decrease in allogeneic and autologous blood transfusion requirements in patients undergoing total hip arthroplasty. No link between TA and perioperative complications, thromboembolic events, or acute renal failure was found, independent of anticoagulant use. The most effective and safe dosage of TA was reported to be 2,000 mg [21].

Our study found the total drainage amount significantly decreased in patients who received TA. When groups were compared, operative and tourniquet times were found to differ. When the control group was divided into those who required a transfusion and those who did not, the tourniquet time and total drainage amount was found to be higher in the transfusion group, although the difference was not statistically significant. The weight and BMI of patients who received TA were lower than the control group. We observed increased operative times, tourniquet times, and increased transfusion needs in patients with a higher weight and BMI. These findings are coherent with that in the literature [4-5, 20]. We also found that the preoperative hemoglobin levels of patients requiring postoperative transfusions were lower in the control group. According to these findings, prolonged tourniquet and operative times, preoperative low hemoglobin level, high BMI, and the use of antifibrinolytic agents have a significant effect on postoperative blood loss and requirement for transfusion.

Retrospective design and the low number of patients are the most important limitations of this study. The low number of patients is due to our inclusion criteria of patients undergoing total knee arthroplasty under spinal anesthesia, although this was required to prevent bias. We did not use any laboratory data nor did we use any clinical test to determine patients with an increased risk of subclinical venous thromboembolism. We did not perform any test to detect any occult blood loss that could have affected our results. It should also be noted that all patients requiring a blood transfusion (n = 11) between postoperative sixth and 24th hours were in the control group and that 24th-hour hemoglobin levels of these patients (11/28) were obtained after transfusions.

## Conclusions

In conclusion, a single and low dose of TA in total knee arthroplasties is an effective and safe agent that significantly decreases the visible blood loss and requirement for allogeneic blood transfusion. Further studies are needed to establish efﬁcacy of different doses of TA with regard to diminishing invisible blood loss, reducing hospital stay, and complication rates related to anesthesia and/or surgical procedures. Otherwise, cost-effectivity studies are required in TA used in different procedures.
